# The Epidemiologic and Clinical Characteristics of the 2023 Dengue Outbreak in Bangladesh

**DOI:** 10.1093/ofid/ofae066

**Published:** 2024-02-02

**Authors:** Nadim Sharif, Nazmul Sharif, Afsana Khan, Shuvra Kanti Dey

**Affiliations:** Department of Microbiology, Jahangirnagar University, Savar, Dhaka, Bangladesh; Department of Mathematics, Rajshahi University of Engineering & Technology, Rajshahi, Bangladesh; Department of Statistics, Jahangirnagar University, Savar, Dhaka, Bangladesh; Department of Microbiology, Jahangirnagar University, Savar, Dhaka, Bangladesh

**Keywords:** Bangladesh, dengue, epidemiology, outbreak, clinical characteristics

## Abstract

The ongoing 2023 dengue outbreak is the worst ever case reported in Bangladesh. There is a lack of epidemiological studies on the outbreak. A 2-tailed *t* test was performed. Multivariable logistic regression analysis was conducted. We found about 277 801 cases and 1393 deaths from the 2023 dengue outbreak. About 52% of the cases were from outside of Dhaka. The male:female ratio was about 3:2. The highest frequency of cases was found among people aged 19–29 years (28.7%, 79 673 of 277 801; *P* = .001). The overall case fatality rate (CFR) was 0.5%. The highest CFR was found among children aged 0–10 years (12%). Fever (99%) was the most prevalent, followed by joint pain (86%). We found significantly higher odds of fatalities (adjusted odds ratio [aOR], 4.21; 95% CI, 3.93–4.74; *P* = .05), cases (aOR, 3.85; 95% CI, 3.25–4.12; *P* = .001) and hospitalizations (aOR, 3.26; 95% CI, 3.11–4.04; *P* = .006) during the 2023 outbreak compared with previous outbreaks during 2008–2022. This is one of the early reports of epidemiological and clinical characteristics of ongoing dengue outbreak.

Dengue is an acute febrile viral disease and has spread to more than 120 countries worldwide [1]. Every year an estimated 400 million cases of dengue occur, with majority of them in the urban and semiurban areas of tropical and subtropical regions [1]. About half of the world population is at risk of getting dengue infection. Dengue virus (DENV) is transmitted to humans by bites of infected *Aedes* spp. mosquitoes [1, 2]. According to the World Health Organization (WHO), symptomatic dengue virus infections can be grouped into 3 major categories: undifferentiated fever, dengue fever (DF), and dengue hemorrhagic fever (DHF) [1, 2]. The majority of people develop asymptomatic infection. In symptomatic cases, clinical symptoms including high fever (104°F), severe headache, pain behind the eyes, muscle and joint pains, nausea, vomiting, swollen glands, and rash are mostly reported [1–4]. In acute cases, severe abdominal pain, persistent vomiting, rapid breathing, bleeding gums or nose, fatigue, restlessness, blood in vomit or stool, being very thirsty, pale and cold skin, and feeling weak are the most commonly reported symptoms [1, 2]. This study aims to provide a descriptive overview of the epidemiological and clinical characteristics of the 2023 dengue outbreak in Bangladesh.

Data were collected from the national dengue surveillance system, Information System (MIS) unit of the Directorate General of Health Services (DGHS) in Bangladesh [3]. Percentage, rate, and frequency were used for representing the categorical variables. Mean and standard deviation were used for representing the central tendency of continuous variables. A 2-tailed *t* test was performed. Multivariable logistic regression analysis was conducted for different factors in the 2023 outbreak and 2008–2022 outbreaks. All of the statistical analyses were performed by IBM SPSS Statistics for Windows (version 28.0; IBM Corp., Chicago, IL, USA) and Microsoft Excel 2021.

Bangladesh is an endemic region for dengue fever, with the first report dating back to 2000. Regular outbreaks of dengue have been reported in Bangladesh since 2000 [4, 5]. As of 2022, about 242 919 cases and 837 fatalities, with 34 deaths per 1000 cases, have been documented [4–6]. About 100% of these cases are hospital tested [5].

The 2023 outbreak started in January 2023, with about 2000 documented cases and 15 deaths per day since the last week of July, which has broken all the previous records of dengue cases in Bangladesh (Supplementary Tables 1 and 2, Supplementary Figure 1) [3–6]. About 277 801 hospitalized cases and 1393 deaths have been recorded as of November 3, 2023, which is the highest number in a single year in Bangladesh (Supplementary Figure 2) [3–9]. As the surveillance included only cases reported in government-approved hospitals, we noticed a significant underreporting of cases of dengue. At present, the estimated number of dengue cases will be 4 times greater than the reported number. A major outbreak with >100 000 hospitalized cases was reported back in 2019 [4–6]. During the peaks of the coronavirus disease 2019 pandemic (2020–2022), reported cases of dengue declined significantly in Bangladesh. Another interesting feature of the 2023 outbreak is a more rapid spread of a large number of dengue cases all over the 64 districts than before. During 2000–2018, Dhaka, the capital of Bangladesh, was the main epicenter of dengue outbreaks, with 90% of cases and fatalities. However, during the 2023 outbreak, about 63.4% (177 084 of 277 801) of dengue cases were found outside Dhaka (23°45′50″N 90°23′20″E). Further, the majority of the deaths (826 of 1393) have been documented in Dhaka. Though the number of cases started to increase after April in Dhaka, it started to spread rapidly outside Dhaka after July 2023. Chattogram (22°20′06″N 91°49′57″E) division (13.2% of cases and 9.2% of deaths) and Barisal division (10.5% of cases and 4.3% of deaths) became 2 hotspots of dengue transmission in 2023. The ongoing outbreak started about 7 months earlier than previous outbreaks. As a result, the highest numbers of cases and deaths were recorded in July and August 2023.

The ratio of male:female was 3:2 among dengue-infected people. The highest frequency of cases was found among people aged 19–29 years (28.7%, 79 673 of 277 801; *P* = .001), followed by 0–18 years (28.1%, 78 181 of 277 801; *P* = .001), 40–59 years (19.5%, 54 067 of 277 801; *P* = .005), 30–39 years (18.2%; 50 423 of 277 801; *P* = .004), 60–79 years (5.2%, 14 560 of 277 801; *P* = .001), and >80 years (0.3%, 897 of 277 801; *P* = .003) (Table 1A). We found a relatively higher frequency of cases among people aged 20–40 years compared with the previous data in Bangladesh [5–9].

**Table 1.** Age and Sex Distribution During the 2023 Dengue Outbreak in Bangladesh

A, Confirmed Cases				
Age Group in Years	Male	Female	Total No. (%)	*P* Value
0–18	49 060 (17.7)	29 121 (10.4)	78 181 (28.1)	.001
19–29	50 960 (18.3)	28 713 (10.4)	79 673 (28.7)	.001
30–39	28 666 (10.3)	21 757 (7.9)	50 423 (18.2)	.004
40–59	29 030 (10.4)	25 037 (9.1)	54 067 (19.5)	.005
60–79	8725 (3.1)	5835 (2.1)	14 560 (5.2)	.001
>80	539 (0.2)	358 (0.1)	897 (0.3)	.003
Total No. (%)	166 980 (60.1)	110 821 (39.9)	277 801 (100)	.005

B, Associated Deaths				
Age Group in Years	Male (n = 598)	Female (n = 795)	Total No. (%) (n = 1393)	*P* Value
0–18	104 (7.5)	110 (7.9)	214 (15.4)	.05
19–29	91 (6.5)	149 (10.7)	240 (17.2)	.002
30–39	75 (5.4)	181 (13.0)	256 (18.4)	.001
40–59	179 (12.8)	253 (18.2)	432 (31.0)	.005
60–79	126 (9.0)	94 (6.7)	220 (15.7)	.001
>80	23 (1.7)	8 (0.6)	31 (2.3)	.01
Total No. (%)	598 (42.9)	795 (57.1)	1393 (100)	.001

A 2-tailed *t* test was performed. A *P* value ≤.05 was statistically significant. The level of significance was .05.

The highest frequency of fatalities was found among people aged 40–59 years (31%, 432 of 1393; *P* = .005), followed by 30–39 years (18.4%, 256 of 1393; *P* = .001), 19–29 years (17.2%, 240 of 1393; *P* = .002), 60–79 years (15.7%, 220 of 1393; *P* = .001), and 0–18 years (15.4%, 214 of 1393; *P* = .05) (Table 1B). The highest case fatality rate (CFR) has been documented among children aged 0–10 years (12%), followed by patients aged >80 years (5.36%), 60–79 years (1.35%), and 40–59 years (0.66%). Further, we found a significantly higher CFR in females than in males (0.57% vs 0.43%; *P* = .001). Further, females have a 3-times-higher CFR than males in the age group 30–39 years (0.72% vs 0.28%). Individuals aged >80 years (5.36%) and children aged <10 years (12%) have a significantly higher CFR compared with the 40–59 years age group (0.66%). The overall CFR and number of fatalities in 2023 are the highest compared with any other previous records in Bangladesh (Supplementary Figure 1) [5–9].

Among the documented clinical symptoms of 47 854 positive cases by NS1 test, fever (99%) was the most prevalent, followed by joint pain (86%), fatigue (86%), malaise (81%), headache (81%), weakness (76%), abdominal cramps (75%), muscle pain (71%), diarrhea (65%), and vomiting (62%). Among the patients with fatalities (310 fatalities), plasma leakage was the most common symptom (74%), followed by multi-organ damage (67%), dengue shock syndrome (13%), and dengue encephalitis (5%). However, appearance of rash had a lower frequency (<10%). Unlike previous outbreaks, about 30%–40% of the patients experience fever lasting only 24–48 hours. In some cases, sudden death after 3–5 days of symptoms has been found. These findings are supported by previous reports in Bangladesh [10–13]. However, changes in several symptoms have been recorded in this outbreak, which will require extensive clinical investigation.

In multivariable regression analysis, we found significantly higher odds of fatalities (adjusted odds ratio [aOR], 4.21; 95% CI, 3.93–4.74; *P* = .05), cases (aOR, 3.85; 95% CI, 3.25–4.12; *P* = .001), and hospitalizations (aOR, 3.26; 95% CI, 3.11–4.04; *P* = .006) during the 2023 outbreak compared with previous outbreaks during 2008–2022 (Table 2). Among the reported clinical symptoms, higher odds of dengue shock syndrome (aOR, 3.31; 95% CI, 3.19–4.02; *P* = .05), plasma leakage (aOR, 2.81; 95% CI, 2.48–3.15; *P* = .01), multi-organ damage (aOR, 2.14; 95% CI, 1.83–2.67; *P* = .01), diarrhea (aOR, 1.96; 95% CI, 1.53–2.41; *P* = .002), and vomiting (aOR, 1.72; 95% CI, 1.46–2.31; *P* = .001) were found during 2023 outbreak. We found lower odds of symptoms including rash (aOR, 0.2; 95% CI, 0.1–0.78; *P* = .05), joint/muscle pain (aOR, 0.28; 95% CI, 0.11–0.82; *P* = .003), and malaise (aOR, 0.33; 95% CI, 0.14–0.95; *P* = .05) among the patients (Table 2).

**Table 2.** Multivariable Regression Analysis on Different Outcomes of Dengue Outbreaks in Bangladesh

Factors	2023 Outbreak vs 2008–2022 Outbreaks, aOR (95% CI)	*P* Value
Cases	3.85 (3.25–4.12)	.001
Fatalities	4.21 (3.93–4.74)	.05
Hospitalization	3.26 (3.11–4.04)	.006
Widespread transmission	2.15 (1.87–2.71)	.005
Clinical symptoms		
Joint/muscle pain	0.28 (0.11–0.82)	.003
Malaise	0.33 (0.14–0.95)	.05
Rash	0.2 (0.1–0.78)	.05
Diarrhea	1.96 (1.53–2.41)	.002
Vomiting	1.72 (1.46–2.31)	.001
Plasma leakage	2.81 (2.48–3.15)	.01
Multi organ damage	2.14 (1.83–2.67)	.01
Dengue shock syndrome	3.31 (3.19–4.02)	.05
Dengue encephalitis	1.24 (1.07–1.63)	.05
Sudden death within 3–5 d of illness	2.48 (2.26–3.16)	.005

Abbreviation: aOR, adjusted odds ratio.

The highest prevalence of cumulative dengue cases was found in the month of August (31.9%, 70 294 of 220 572), followed by September (19.1%, 42 223 of 220 572), October (18.9%, 41 729 of 220 572), and November (13.7%, 30 155 of 220 572), during 2008 to 2022 (Supplementary Table 3). The peak of dengue (83% cases) has historically been confined within 4 months from August to November in Bangladesh. Since 2016, dengue fever has been present all throughout the year. However, the highest prevalence was found to be in August in 2008 (473 cases), 2010 (183 cases), 2011 (691 cases), and 2019 (52 636 cases) and in September in 2009 (188 cases), 2012 (246 cases), 2015 (965 cases), 2016 (1544 cases), 2018 (3087 cases), and 2021 (7841 cases). During the ongoing 2023 outbreak, we found the highest number of cases in every month compared with previous years in Bangladesh (Supplementary Table 3). The highest incidence was found in September (about 80 000 cases), followed by August (about 72 000 cases) (Supplementary Figure 2). The peak of the ongoing outbreak is predicted to be extending longer than any other previous outbreaks in Bangladesh. These seasonal data are similar to previous studies in Bangladesh and other countries where monsoon and postmonsoon outbreaks occur [4, 5, 7, 9, 11, 12].

Climate change, mass migration, urbanization, and environmental pollution are contributing significantly to increased concentration of dengue vectors, namely *Aedes* spp. [13–16]. Previously it was well known that *Aedes* spp. require clean and fresh water for reproduction. However, recently it has been found that *Aedes* spp. can also use drain and wastewater for larval growth [14, 15]. Recently, it has been also found that *Aedes* spp. can bite both during the day and night [14, 16]. This changing behavior of vectors, along with climate changes and human activities, is contributing to larger and more unusual outbreaks of dengue in Bangladesh. Further, genotypic characterization of dengue virus is minimal in Bangladesh. DENV2 was the most commonly reported genotype until 2018 [1, 8]. However, DENV3 replaced DENV2 after 2018. Among 66 samples analyzed in June 2023, DENV2 (51.5%) was the predominant genotype, followed by DENV3 (43.9%). This study has number of limitations. As the data are from hospital-reported cases only, there may be underreporting of cases. Further, we could not add data on seroprevalence as we had no serological data.

This is one of the early epidemiological and clinical studies characterizing the ongoing 2023 dengue outbreak. The data in this study will add knowledge for future broad studies to investigate the dengue outbreaks in Bangladesh.

## Supplementary Material

ofae066_Supplementary_Data

## Supplementary Data

Supplementary materials are available at *Open Forum Infectious Diseases* online. Consisting of data provided by the authors to benefit the reader, the posted materials are not copyedited and are the sole responsibility of the authors, so questions or comments should be addressed to the corresponding author.
