# Supplementary material for: The Epidemiologic and Clinical Characteristics of the 2023 Dengue Outbreak in Bangladesh
Source: Open Forum Infect Dis. 2024 Feb 2;11(2):ofae066. doi: 10.1093/ofid/ofae066 (PMC10883285; doi:10.1093/ofid/ofae066)
Supplement: ofae066_Supplementary_Data [file ofae066_supplementary_data.docx]

**Supplementary Table 1.** Probable per-day scenario of the outbreak from July to November, 2023 in Bangladesh.

| **Places** | **Positive**  **Cases/day** | **Hospitalized**  **Cases/day** | **Discharged/day** | **Death/day** |
| --- | --- | --- | --- | --- |
| **Inside Dhaka City** | 853 | 11 | 836 | 6 |
| **Outside Dhaka City** | 1293 | 24 | 1266 | 3 |
| **Total cases** | 2146 | 35 | 2102 | 9 |

**Supplementary Table 2.** Distribution of dengue cases, hospitalized patients and dead patients inside the Dhaka city and outside of the city from 01 January to 03 November 2023.

| **Places** | **Positive**  **cases** | **Non-hospitalized** | **Death** | **Hospitalized** | **Percentage death** |
| --- | --- | --- | --- | --- | --- |
| **Dhaka** | 100717 | 98054 (92) | 826 | 1837 (8) | 0.8 |
| **Outside Dhaka city** | 177084 | 171718 (91) | 567 | 4799 (8) | 0.3 |
| **Total** | 277801 | 269772 (91) | 1393 | 6636 (8) | 0.5 |

**Supplementary Table 3.** Monthly distribution of dengue cases from 2008 to 2023 in Bangladesh.

| **Month** | **2008** | **2009** | **2010** | **2011** | **2012** | **2013** | **2014** | **2015** | **2016** | **2017** | **2018** | **2019** | **2020** | **2021** | **2022** | **Total Cases** | **% Cases** | **2023** |
| --- | --- | --- | --- | --- | --- | --- | --- | --- | --- | --- | --- | --- | --- | --- | --- | --- | --- | --- |
| January | 0 | 0 | 0 | 0 | 0 | 6 | 15 | 0 | 13 | 92 | 26 | 38 | 199 | 32 | 126 | 547 | 0.25 | 566 |
| February | 0 | 0 | 0 | 0 | 0 | 7 | 7 | 0 | 3 | 58 | 7 | 18 | 45 | 9 | 20 | 174 | 0.08 | 166 |
| March | 0 | 0 | 0 | 0 | 0 | 3 | 2 | 2 | 17 | 36 | 19 | 17 | 27 | 13 | 20 | 156 | 0.07 | 111 |
| April | 0 | 0 | 0 | 0 | 0 | 3 | 0 | 6 | 38 | 73 | 29 | 58 | 25 | 3 | 23 | 258 | 0.12 | 143 |
| May | 0 | 1 | 0 | 0 | 0 | 12 | 8 | 10 | 70 | 134 | 52 | 193 | 10 | 43 | 163 | 696 | 0.32 | 1036 |
| June | 0 | 0 | 0 | 61 | 10 | 50 | 9 | 28 | 254 | 267 | 295 | 1884 | 20 | 272 | 737 | 3887 | 1.76 | 5956 |
| July | 160 | 4 | 61 | 255 | 129 | 172 | ***82*** | 171 | 926 | 286 | 946 | 16,253 | 23 | 2286 | 1571 | 23,325 | 10.57 | 43854 |
| August | ***473*** | 125 | ***183*** | ***691*** | 122 | 339 | 80 | 765 | 1451 | 346 | 1796 | ***52,636*** | 68 | 7698 | 3521 | ***70,294*** | 31.87 | 71976 |
| September | 334 | ***188*** | 120 | 193 | ***246*** | 385 | 76 | ***965*** | ***1544*** | 430 | ***3087*** | 16,856 | 47 | ***7841*** | 9911 | ***42,223*** | 19.14 | ***79598*** |
| October | 184 | 154 | 45 | 114 | 107 | ***501*** | 63 | 869 | 1077 | ***512*** | 2406 | 8143 | 164 | 5458 | ***21,932*** | ***41,729*** | 18.92 | 67769 |
| November | 0 | 0 | 0 | 36 | 27 | 218 | 22 | 271 | 522 | 409 | 1192 | 4011 | ***546*** | 3567 | 19,334 | 30,155 | 13.67 | 6626 |
| December | 0 | 0 | 0 | 9 | 0 | 53 | 11 | 75 | 145 | 126 | 293 | 1247 | 231 | 1207 | 3731 | 7128 | 3.23 |  |
| Total | **1151** | **472** | **409** | **1359** | **641** | **1749** | **375** | **3162** | **6060** | **2769** | **10148** | **101354** | **1405** | **28429** | **61089** | **220572** | **100** | **277801** |

Italic bold indicated the highest number of cases in a single year.

**Figure 1.** Yearly distribution of cases and fatalities of dengue from 2000 to 2023 in Bangladesh. The data are added till 04 November, 2023.

**Figure 2.** Monthly distribution of cases and fatalities of 2023-dengue outbreak in Bangladesh.
